# Still seeing little organizational diversity: the long-run outcomes of market-inspired reforms in Swedish primary care

**DOI:** 10.1108/JHOM-02-2026-0184

**Published:** 2026-05-20

**Authors:** Peter Edlund, Alfons Grönström, Ulrika Winblad

**Affiliations:** Department of Public Health and Caring Sciences, Uppsala University, Uppsala, Sweden

**Keywords:** Sweden, Scandinavia, Primary care, Organizational diversity, Private providers, Market-inspired reforms

## Abstract

**Purpose:**

Our aim throughout this article is to develop a comprehensive description detailing the organizational diversity existing among private providers of primary care services in Sweden today.

**Design/methodology/approach:**

We begin by mapping all public and private providers in Swedish primary care during 2024. We continue by examining the organizational diversity existing across three salient management dimensions – business types, core purposes and external funding sources – among all private providers, which represented ≈47% of all primary care centers and ≈44% of all primary care patients.

**Findings:**

Concerning business types, we find that most private providers functioned as private limited companies, which commanded 80% of all privately operated primary care centers and ≈75% of all privately listed primary care patients. Regarding core purposes, we find that most private providers conducted for-profit activities, which affected ≈95% of all privately operated centers and privately listed patients. And, concerning external funding sources, we find that most private providers utilized credit institute loans, which impacted ≈80% of all privately operated centers and ≈75% of all privately listed patients.

**Originality/value:**

Our findings contribute to previous research on private providers of primary care services in Sweden by offering an updated and expanded description highlighting how we are “still seeing little organizational diversity” among said providers. These findings are problematic for politicians across Scandinavia, considering they have, ever since the early 1990s, sought to attract diverse private actors as providers of public sector services.

## Introduction: launching public sector reforms to attract a diversity of private actors

Since the early 1990s onward, politicians across Scandinavia have recurrently launched reforms attempting to create market-like conditions among providers of public sector services (Knutsson *et al.*, 2016; Svallfors and Tyllström, 2019; Vike *et al.*, 2024). Sweden has moved at “the forefront of this wave of experiments” (Andersson *et al.*, 2021, p. 85), and its reforms have been more persistent than anywhere else throughout the Scandinavian countries. During the past 35 years or so, Swedish politicians have sought cost savings for governments and quality improvements for users by persistently launching market-inspired reforms in attempts to attract a diversity of private actors into public sector services (Andersson *et al.*, 2023; Blomqvist and Rothstein, 2000; Wisell *et al.*, 2019).

Reforms tend to be delicate projects, however, and the outcomes politicians intended are not always realized. Instead, reforms usually generate a blend of intended and unintended outcomes (Brunsson, 1989, 2009; Olsen, 1991). It is clear that Sweden's market-inspired reforms have succeeded in attracting privately owned actors as providers of diverse services across the public sector. Private providers increased their operations from supplying almost no public services throughout the early 1990s to supplying services worth more than 20% of total turnover generated by all public services during 2022 (SCB, 2024). That said, it is unclear whether Sweden's reforms have succeeded in attracting a diversity of privately owned providers – such as companies and foundations, or for-profit and not-for-profit providers – to the public sector. This is a pressing matter, as politicians have repeatedly argued that attracting diverse private providers remains essential to foster competition and choice (Andersson *et al.*, 2023; Blomqvist and Rothstein, 2000; Wisell *et al.*, 2019), mechanisms that are, in turn, expected to engender cost savings and quality improvements throughout public services (Ehn and Sundström, 2020; Hartman, 2011).

Researchers examining market-inspired reforms throughout the Swedish public sector have often turned toward primary care, which is a particularly fruitful context to study these reforms because all county councils (nowadays labeled regions) across Sweden must, since 2010, facilitate competition and choice by welcoming private actors as providers of primary care services. Primary care has, in this sense, become one of the most attractive public sector areas for privately owned providers (Vengberg *et al.*, 2021; Winblad *et al.*, 2021). Researchers have passingly highlighted that diversity among private primary care providers may be expressed through differences in core purposes, which are associated with the most central intention issues of conducting for-profit or not-for-profit activities (Häger Glenngård, 2023; Isaksson *et al.*, 2016; Vengberg *et al.*, 2021; Winblad *et al.*, 2021). Although core purposes have not been in focus, previous research on Swedish primary care suggests there was little diversity among privately owned providers throughout the 2010s, considering almost all these providers conducted for-profit activities. Distinguishing between for-profit and not-for-profit activities as two core purposes is nonetheless relevant because they reflect fundamentally different goals shaping the ways that organizations can be managed: to prioritize economic benefit or to prioritize social impact.

Corporate governance researchers have underscored how core purposes constitute an important management dimension – that is, a critical aspect significantly influencing the priorities of managers leading organizations (Aguilera and Crespi-Cladera, 2016; Santos *et al.*, 2024). However, research on corporate governance suggests management dimensions beyond core purposes may also influence the ways that private providers are managed in Sweden's primary care. Studies show that multiple management dimensions – core purposes as well as business types (Aguilera and Crespi-Cladera, 2016) and external funding sources – can simultaneously influence the priorities of managers at privately owned providers. Business types are associated with ownership issues; the absence or presence of shareholders as owners, for example, affects to whom private providers are accountable. External funding sources are, moreover, associated with financing issues; the support of certain financiers may, for instance, offer more autonomy to privately owned providers than the support of other financiers. This, by extension, implies that a single management dimension is insufficient for scholars to grasp the diverse ways that private providers can be managed in Swedish primary care. A multidimensional approach will not only advance research on privately owned providers of primary care services in Sweden but also help politicians make informed assessments about how private actors, through their management dimensions, can be expected to operate as public service providers.

Throughout this article, we embark on an initial, yet crucial, endeavor to enhance extant scholarly understanding of organizational diversity – here approached as the differences across management dimensions among organizations (cf. Wisell *et al.*, 2019) – in Swedish primary care. Our aim is to develop a comprehensive description detailing the organizational diversity existing among privately owned providers of primary care services in Sweden today. We address this aim by first mapping all public and private providers operating in Swedish primary care during 2024, and then examining organizational diversity across three salient management dimensions – business types, core purposes and external funding sources – among all private providers. Our reasoning for starting with business types is that they must be identified before we can proceed with core purposes and external funding sources.

In brief, three main findings emerge from our mapping and examining of 2024. Concerning business types, most privately owned providers operated as private limited companies. Regarding core purposes, we found that a majority of private providers conducted for-profit activities. And, concerning external funding sources, most privately owned providers utilized credit institute loans. Altogether, our findings indicate we are “still seeing little organizational diversity” among private providers of primary care services, even though increasing such diversity has, since the early 1990s, been a key policy target for Sweden's public sector at large. These findings advance previous research on privately owned providers in Swedish primary care by offering a significantly updated and expanded description highlighting the little organizational diversity existing today among said providers. Our findings can also be fruitfully leveraged beyond Sweden to grasp the characteristics of private providers nowadays operating throughout other Scandinavian countries with large public sectors. We ultimately discuss our findings by drawing on classical insights from institutional theory (e.g. DiMaggio and Powell, 1983; Meyer and Rowan, 1977). In doing so, we point to business types other than companies, to political debates about dominant core purposes, and to private equity investments as relatively uncommon external funding sources among private providers of primary care services that nevertheless display isomorphic tendencies (cf. (Boxenbaum and Jonsson, 2017; DiMaggio and Powell, 1983), when approximately 35 years have transpired since market-inspired reforms began sweeping across Scandinavia.

## Literature review: crafting a framework to analyze organizational diversity

Privately owned providers are present in considerable numbers throughout the Scandinavian countries, supplying a substantial percentage of public sector services (Knutsson *et al.*, 2016; Svallfors and Tyllström, 2019; Vike *et al.*, 2024). Yet researchers studying such services have “consistently treated private providers as one, homogenous, group” (Andersson *et al.*, 2021, p. 86). Treating these providers as a homogenous group risks bypassing the potential existence of organizational diversity among them, expressed through important differences in how privately owned providers are managed.

As concept, organizational diversity derives from workforce management literature, where such diversity has most often concerned the notion of employees displaying various backgrounds and commanding various competencies (e.g. Jackson *et al.*, 2003; Van de Ven *et al.*, 2008). In health services research, however, organizational diversity has instead come to denote the notion of care providers presenting various sizes and structures (Wisell *et al.*, 2019). This latter notion of organizational diversity, with its focus on organizational size and structure, is closely related to our aim.

Previous research highlighting organizational diversity among private providers in public sectors across Scandinavia is scant, however. Most of this research comes from scholars who, while studying other topics, have highlighted the ways that organizational diversity is expressed through core purposes among privately owned providers in Swedish primary care. For instance, when studying reimbursement schemes with data from 2016, Vengberg *et al.* (2021, p. 461) claimed that the core purposes of private providers supplying primary care services in Sweden “almost exclusively” encompassed conducting for-profit activities. Similar claims have been made *en passant* by several researchers utilizing data from the 2010s to study other topics than core purposes among privately owned providers in Swedish primary care (Häger Glenngård, 2023; Isaksson *et al.*, 2016; Winblad *et al.*, 2021).

Some researchers have nonetheless ventured further to highlight more than core purposes. For example, when studying governance modes with data from 2014, Angelis *et al.* (2016) meshed core purposes and business types to create two categories of private providers supplying primary care services in Sweden. In this categorization, for-profit “Private companies” supplied more than 95% of all privately provided primary care services, while not-for-profit “Foundations and economic associations” supplied less than 5% (Angelis *et al.*, 2016, p. 25). Moreover, when studying quality perceptions with data from 2017, Andersson *et al.* (2021) meshed core purposes and business types to create four categories of private providers in Swedish primary care. In this categorization, “Company group, for profit” supplied more than 60% of all privately provided primary care services; “Independent units, for profit” close to 35%; and “Company group, not for profit” and “Independent units, not for profit” less than 5% each (Andersson *et al.*, 2021, p. 88). That said, Andersson *et al.* (*ibid*) also acknowledged how “the distinction between the categories is not always entirely clear,” and these researchers ultimately utilized core purposes as a baseline when distinguishing between the four categories of private providers.

**Figure 1:**
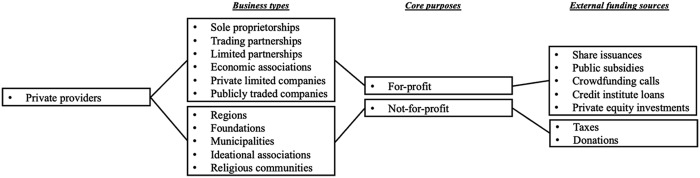
Analytical framework featuring three management dimensions influencing the priorities of managers leading private providers supplying primary care services in Sweden. Source: Authors' own work

Whereas previous research focusing on privately owned providers supplying primary care services in Sweden offers plenty of insights into organizational diversity as expressed through core purposes, insights remain sporadic and unsystematic when it comes to such diversity as expressed through business types. In relation to business types, Janlöv *et al.* (2023, p. 114) even suggested that “national statistics on the ownership structure of primary care providers is not available.” Our argument is that previous research can be advanced by categorizing private providers of primary care services differently – and more clearly – than they have hitherto been categorized. To advance this research, we draw inspiration from corporate governance scholars who indicate private actors may be fruitfully categorized along the lines of business types, core purposes and external funding sources as three salient dimensions affecting what priorities managers make when leading organizations (Aguilera and Crespi-Cladera, 2016; Santos *et al.*, 2024). Our study aligns with these dimensions, and we transpose their specific contents into a customized framework to analyze the presence of diverse business types, core purposes, and external funding sources among privately owned providers in Swedish primary care. This analytical framework is pictured in Figure 1.

### Empirical background: placing organizational diversity amid Sweden's repeated reforms

To analyze organizational diversity in management dimensions among private providers of primary care services, we begin by situating our study amid Swedish public sector reforms that have been launched repeatedly from the early 1990s onward. Before these reforms, next to no privately owned providers operated in Sweden. Attracting diverse private actors as providers of public sector services has been a key policy target throughout the reforms in question.

Already in 1982, politicians launched a “Health and Medical Services Act” (*Swe.* Hälso-och sjukvårdslagen) that decentralized most responsibility for healthcare services, including primary care, from the Swedish state to Sweden's 23 county councils (Häger Glenngård *et al.*, 2005). Such decentralization was a necessary precondition for the subsequent launch of an “Act on Public Procurement” (*Swe.* Lagen om offentlig upphandling) in 1992. Often dubbed the “LOU” law, this act made it possible for politicians to introduce tender procedures through which county councils could contract certain public services, like primary care, among publicly owned and/or privately owned providers on cost and quality grounds (Anell, 2011).

The LOU law can be interpreted as an explicit effort by Swedish politicians to create local markets based on competition and choice in primary care services, among others. And this effort successively generated an impact in terms of attracting private providers. By 1995, more than 50% of all county councils in Sweden were contracting primary care services among privately owned providers, although the absolute numbers of such providers remained small (Häger Glenngård *et al.*, 2005). Throughout the late 1990s and early 2000s, growing numbers of county councils adopted LOU-associated tender procedures, often to facilitate so-called purchaser–provider systems (Dahlgren, 2018). These procedures had, by the mid-2000s, been adopted in 90% of all county councils (Häger Glenngård *et al.*, 2005). Most importantly, LOU-associated procedures fueled a gradual growth of private providers; in 2007, they operated 26% of all primary care centers (Janlöv *et al.*, 2023).

Reaching 2009, Swedish politicians launched an “Act on System of Choice in the Public Sector” (*Swe.* Lagen om valfrihetssystem)—often labeled the “LOV law” –, and it can be interpreted as an additional effort to create local markets in, among others, primary care services. Unlike the LOU law, however, county councils were mandated to implement the LOV law. And, also unlike the LOU law, county councils were, through the LOV law, expected to welcome and accept service offers from publicly and/or privately owned providers without arranging tender procedures. Providers could thus quickly commence operating (Winblad *et al.*, 2021). The LOV law was ultimately meant to augment organizational diversity by attracting private actors that had not achieved any significant presence as providers of public services, including foundations and religious communities (Wisell *et al.*, 2019). By 2015, publicly and privately owned providers of primary care services were – to different degrees – operating alongside one another in all county councils. And, entering the 2020s, privately owned providers not only operated in all county councils (which had now been renamed as regions (Endnote i)), but these providers also commanded 44% of all primary care centers (Janlöv *et al.*, 2023). This percentage offers a bridge to our study, where we focus on 2024.

## Methods: studying organizational diversity in Swedish primary care

Positioned 35 years or so after market-inspired reforms were initially launched, our study relies on a quantitative dataset featuring information pertaining to all public and private providers of primary care in Sweden during 2024. We studied this year to produce a description that can advance previous research on organizational diversity among privately owned providers in Swedish primary care. This research features sporadic and unsystematic insights for the 2010s, and insights for the 2020s are barely available at all.

### Material and data collection

To build our dataset, we proceeded in two steps. Our first step was to collect information on the names, addresses, providers and listed patients for all 1,229 public and private primary care centers in operation throughout Sweden during September 2024. There is no national register or inventory for primary care centers, so we gathered information about them by querying all 21 regional websites. Detailed information about 310 primary care centers was missing on 9 region websites, however. For these regions, we contacted 9 region administrators to collect the missing information.

Our second step was to gather information on the business types, core purposes and external funding sources for all private providers, which commanded 580 primary care centers (≈47% of all publicly and privately operated centers) and 4,541,304 primary care patients (≈44% of all publicly and privately listed patients) during September 2024. We collected the focal information on types, purposes, and funding sources by searching through entries and annual reports in Retriever Research's extensive “Company Info” repository as well as by searching through newspaper articles in Retriever Research's equally extensive “Media Archive” repository. These two are the largest repositories for company and media data in Scandinavia.

### Data analysis

In examining the resulting dataset, we moved through three stages based on our analytical framework of multiple management dimensions (Figure 1). First, we coded all privately owned providers following the Swedish Companies Registration Office's list of business types (Bolagsverket, 2021). This list includes regions, foundations, municipalities, sole proprietorships, trading partnerships, limited partnerships, economic associations, ideational associations, religious communities, private limited companies and publicly traded companies as business types. Throughout our first coding stage, we focused on identifying a business type at the highest intra-organizational level (i.e. delivery, parent or group level) for each private provider. Our focus derives from the notion that privately owned providers in Sweden's public sector often feature complex intra-organizational structures (cf. Carlbaum and Rönnberg, 2024). Such complexity implies that a business type identified at the lowest level (i.e. delivery level) may differ from the business types identified at higher levels (i.e. parent or group levels). In focusing on the highest intra-organizational level, we assume that managers working at this level wield significant influence, and that their influence reaches levels further down within organizations (Hambrick, 2007).

Second, after our coding according to business types, we also coded all private providers according to core purposes as identified at the highest intra-organizational level. When coding for core purposes, we approached the operations of sole proprietorships, limited partnerships, trading partnerships, economic associations, private limited companies and publicly traded companies as for-profit activities, while we approached the operations of regions, foundations, municipalities, ideational associations and religious communities as not-for-profit activities. Our assumption is once again that managers at the highest level exert considerable influence within organizations, and that this influence reaches levels further down.

Lastly, we coded all privately owned providers following the Swedish Tax Agency's, the Swedish Companies Registration Office's and the Swedish Agency for Economic and Regional Growth's joint list of external funding sources (Verksamt, 2025). This joint list includes taxes, donations, share issuances, public subsidies, crowdfunding calls, credit institute loans and private equity investments. There is also a similar list of internal funding sources including owner investments, retained resources and asset sales. We bypass internal funding sources because most – if not all – providers (i.e. both public and private) supplying public sector services leverage a mix of owner investments, retained resources, and asset sales to fund operations (Santos *et al.*, 2024). This implies it would be largely redundant to code all private providers in our dataset along the lines of internal funding sources. Coding for external funding sources, our focus was on the highest intra-organizational level because managers at this level not only wield significant influence within organizations, but also because the highest level is usually also where potential instances of private equity investments as an external funding source are located. Such investments give private equity companies, which act as financiers, partial or complete control over other organizations (Barber and Goold, 2007)—for instance, over private providers supplying primary care services in Sweden. Private equity investments merit particular focus because they tend to be regarded as controversial funding sources when Swedish citizens are queried about their perceptions of public sector services (cf. Svallfors and Tyllström, 2019).

Altogether, we leveraged our three coding steps to render a comprehensive description showing the organizational diversity found across multiple management dimensions among privately owned providers of primary care services in Sweden during 2024. We elaborate on our description below, deploying descriptive statistics to display findings for primary care centers and primary care patients as alternative counts and proportions.

### Findings: detailing organizational diversity in Swedish primary care

Analyzing private providers in Sweden's primary care during 2024, we found little organizational diversity as expressed through business types, core purposes and external funding sources.

Our first task, however, is to analyze the intra-organizational structures of privately owned providers. This analysis successively makes it possible to identify patterns of concentration. Analyzing intra-organizational structures reveals that Swedish primary care featured private providers ranging from single-level actors (i.e. only displaying a delivery level) to multi-level actors (i.e. displaying a combination of delivery, parent and group levels). Analyzing intra-organizational structures also reveals that many privately owned providers supplying primary care services at the delivery level – perhaps projecting an appearance as small independent actors – formed part of large conglomerate actors featuring delivery, parent and group levels. Table 1 contains examples of single-level actors as well as of multi-level actors among private providers.

**Table 1:** Examples showing single-level actors as well as multi-level actors among private providers of primary care services in Sweden during 2024

Single-level actors
Delivery level: Hälsingeläkarna
*Multi-level actors*
Delivery level: Sophiahemmet Parent level: Sophiahemmet Group level: Sophiahemmet
Delivery level: Akka Hälsocentral; Berga Läkarhus; Centrumläkarna i Helsingborg; Läkarhuset i Karlshamn; Precare Blekinge; Sickla Hälsocenter; Solklart Vård i Bjuv Parent level: Albertina Group level: Nordstjernan

**Source(s):** Authors' own work

The intra-organizational structures of private providers indicate there existed 200 distinct providers that, on a delivery level, commanded 580 primary care centers (≈47% of all publicly and privately operated centers) and 4,541,304 primary care patients (≈44% of all publicly and privately listed patients). 76 distinct providers were structured as single-level actors commanding 1–3 centers each, altogether supplying primary care services through 80 centers (≈15% of all privately operated centers) with 460,779 patients (≈10% of all privately listed patients). The remaining 124 distinct providers were structured under multi-level actors commanding 1–101 centers each, altogether supplying primary care services through 500 centers (≈85% of all privately operated centers) with 4,080,525 patients (≈90% of all privately listed patients). Table 2 features primary care centers and primary care patients for the 200 distinct providers structured as single-level actors or multi-level actors.

**Table 2:** Primary care centers and primary care patients for the 200 distinct providers structured as single-level actors or multi-level actors among private providers of primary care services in Sweden during 2024. The displayed percentages have been rounded

Providers and levels	Primary care centers (percentages of all private centers)	Primary care patients (percentages of all private patients)
76 distinct providers structured as single-level actors	80 (15)	460,779 (10)
124 distinct providers structured under multi-level actors	500 (85)	4,080,525 (90)
*Total*	*Total*	*Total*
200 distinct entities	580 (100)	4,541,304 (100)

**Source(s):** Authors' own work

**Figure 2:**
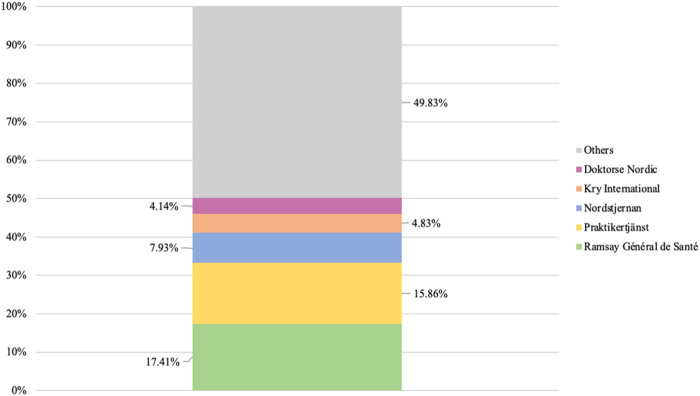
Proportions of all privately operated primary care centers (total = 580) concentrated among the five largest private providers in Sweden during 2024. Source: Authors' own work

**Figure 3:**
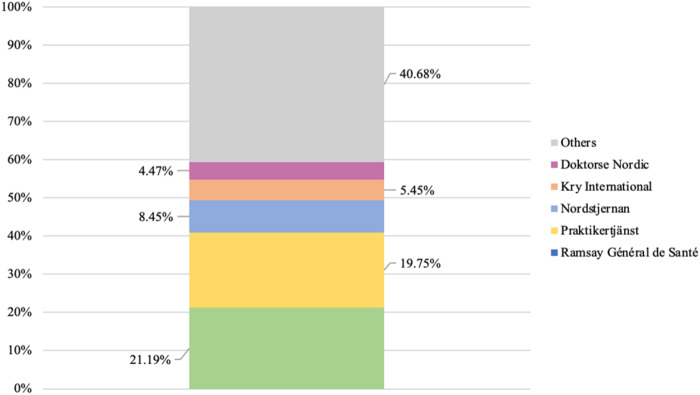
Proportions of all privately listed primary care patients (total = 4,541,304) concentrated among the five largest private providers in Sweden during 2024. Source: Authors' own work

In analyzing multi-level actors featuring delivery, parent and group levels, it is, by extension, possible to detect patterns of concentration among private providers. Such patterns would not be detectable if this analysis were restricted to the delivery level. Figures 2 and 3 display the proportions of all privately operated centers and privately listed patients that were structured under Sweden's five largest private providers, which operated as multi-level actors, during 2024.

As can be seen above, more than 50% of all privately operated primary care centers (Figure 2) and close to 60% of all privately listed patients (Figure 3) were structured under five multi-level actors. These five actors constituted approximately 3% of all private providers. Conversely, this concentration implied that less than 50% of all centers operated by private providers (Figure 2) and just above 40% of all patients listed at private providers (Figure 3) were distributed among 195 single-level actors or multi-level actors. These 195 actors constituted roughly 97% of all private providers.

Having analyzed concentration patterns, we continue by examining what business types, core purposes and external funding sources were present, as well as to what degree they were present, among all private providers in Swedish primary care during 2024.

### Private limited companies as dominant business types

**Figure 4:**
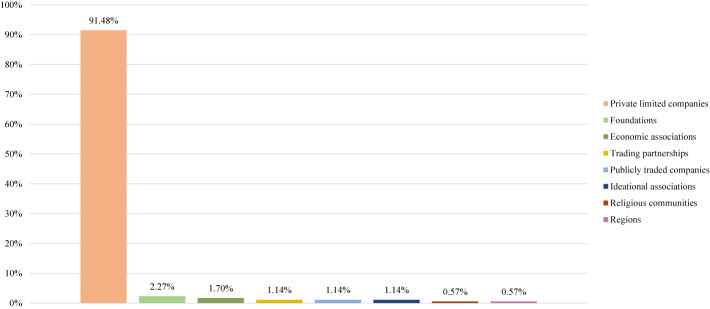
Proportions of business types per all private providers in Swedish primary care during 2024 (total = 200). Source: Authors' own work

**Figure 5:**
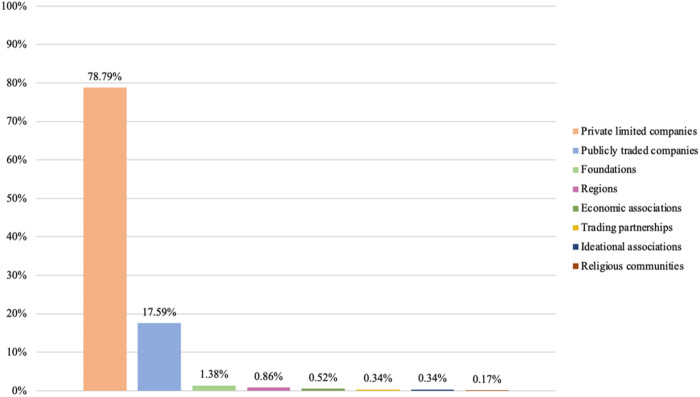
Proportions of business types per all privately operated primary care centers in Sweden during 2024 (total = 580). Source: Authors' own work

**Figure 6:**
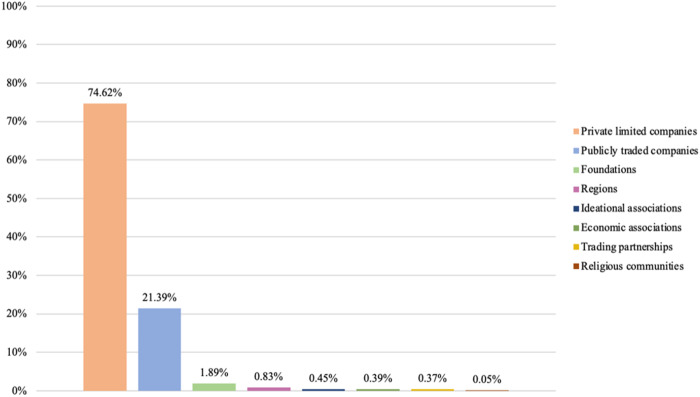
Proportions of business types per all privately listed primary care patients in Sweden during 2024 (total = 4,541,304). Source: Authors' own work

Our next step is therefore to examine the diversity of business types present among privately owned providers. We examine this presence by considering the highest intra-organizational level for each provider. Figures 4–6 depict the proportions of business types among all private providers supplying primary care services in Sweden during 2024.

Examining primary care centers and primary care patients shows that private limited companies constituted the most prevalent business type among private providers. More than 90% of all private providers operated as private limited companies (Figure 4), and they were connected to almost 80% of all privately operated centers (Figure 5) and almost 75% of all privately listed patients (Figure 6). Publicly traded companies formed another business type, although such companies accounted for just over 1% of all private providers (Figure 4). That said, publicly traded companies supplied close to 20% of all privately operated centers (Figure 5) and commanded more than 20% of all privately listed patients (Figure 6). The remaining business types, when aggregated, comprised around 5% of all private providers (Figure 4); they were connected to circa 5% of all privately operated centers (Figure 5) as well as to circa 5% of all privately listed patients (Figure 6).

### For-profit activities as dominant core purposes

**Figure 7:**
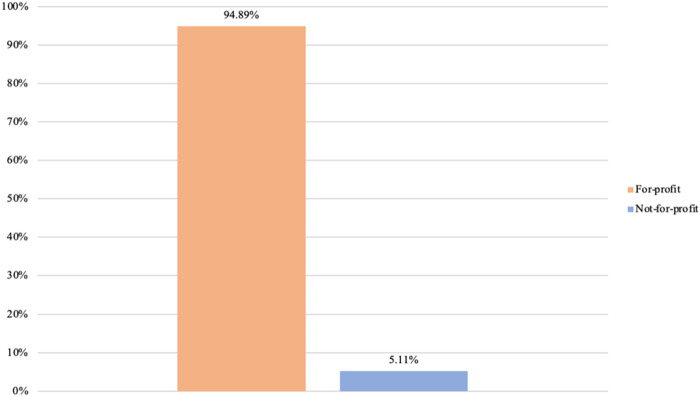
Proportions of core purposes per all private providers in Swedish primary care during 2024 (total = 200). Source: Authors' own work

**Figure 8:**
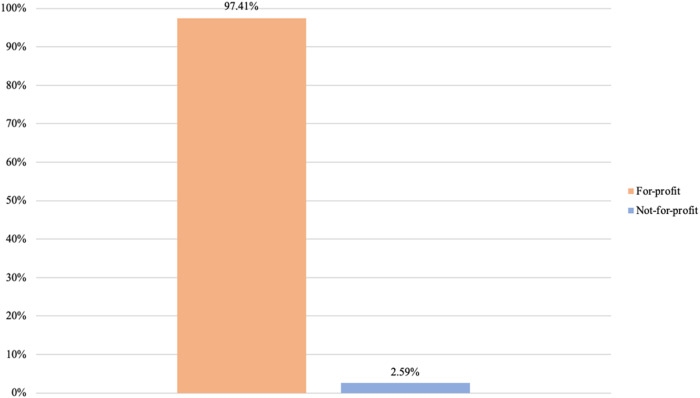
Proportions of core purposes per all privately operated primary care centers in Sweden during 2024 (total = 580). Source: Authors' own work

**Figure 9:**
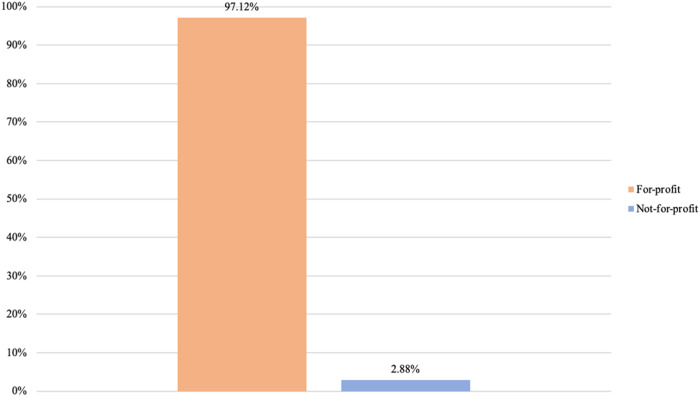
Proportions of core purposes per all privately listed primary care patients in Sweden during 2024 (total = 4,541,304). Source: Authors' own work

Having examined business types, our next step is to analyze the diversity of core purposes present among private providers. Once again, we analyze such presence by considering the highest intra-organizational level for each provider. Figures 7–9 display the proportions of core purposes among all privately owned providers in Swedish primary care during 2024.

Seen in terms of primary care centers and primary care patients, conducting for-profit activities was the most prevalent core purpose among private providers. Almost 95% of all private providers carried out for-profit activities (Figure 7), supplying more than 95% of all privately operated centers (Figure 8) and commanding more than 95% of all privately listed patients (Figure 9). The remaining 5% of all private providers conducted not-for-profit activities (Figure 7), and these private providers encompassed less than 5% of all privately operated centers (Figure 8) and less than 5% of all privately listed patients (Figure 9).

### Credit institute loans as dominant external funding sources

**Figure 10:**
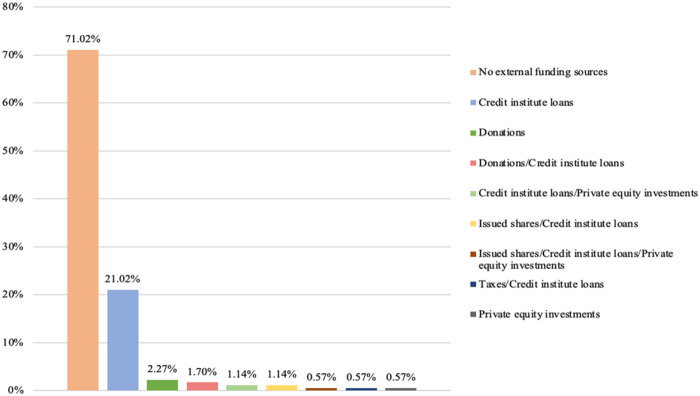
Proportions of external funding sources per all private providers in Swedish primary care during 2024 (total = 200). Source: Authors' own work

**Figure 11:**
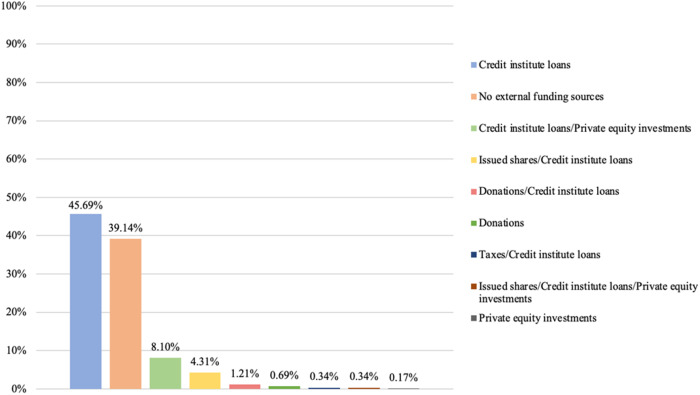
Proportions of external funding sources per all privately operated primary care centers in Sweden during 2024 (total = 580). Source: Authors' own work

**Figure 12:**
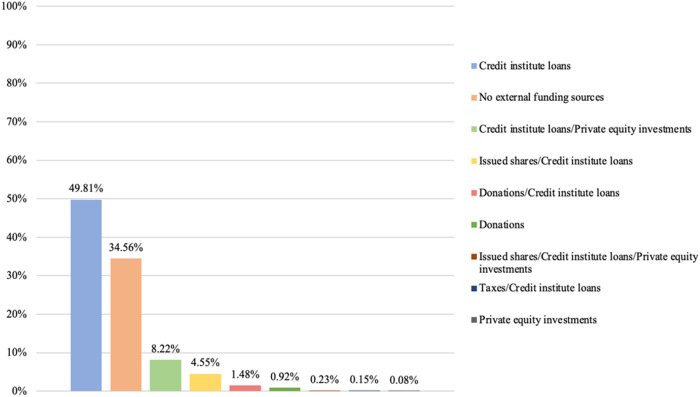
Proportions of external funding sources per all privately listed primary care patients in Sweden during 2024 (total = 4,541,304). Source: Authors' own work

Our final step is to examine the diversity of external funding sources present among private providers. As before, we examine this presence by considering the highest intra-organizational level for each provider. Figures 10–12 depict the proportions of external funding sources among all privately owned providers supplying primary care services in Sweden during 2024.

Examining primary care centers and primary care patients shows that credit institute loans constituted the most utilized external funding source among private providers. More than 20% of all private providers only relied on credit institute loans as external funding (Figure 10), supplying more than 45% of all privately operated centers (Figure 11) and attending close to 50% of all privately listed patients (Figure 12). In addition, just over 1% of all private providers utilized credit institute loans and private equity investment mixes for funding (Figure 10); these private providers were connected to almost 10% of all privately operated centers (Figure 11) as well as to almost 10% of all privately listed patients (Figure 12). And just over 1% of all private providers relied on mixes comprising credit institute loans and issued shares (Figure 10), commanding circa 5% of all focal centers (Figure 11) and circa 5% of all focal patients (Figure 12). More than 10% of all private providers were connected to the remaining external funding sources (Figure 10), and these private providers accounted for less than 5% of all privately operated centers (Figure 11) and for less than 5% of all privately listed patients (Figure 12). However, just over 70% of all private providers refrained from utilizing any external funding (Figure 10); they supplied almost 40% of all focal centers (Figure 11) and attended almost 35% of all focal patients (Figure 12).

At this stage, we pay special attention to private equity investments as external funding sources. During 2024, four private providers (=2% of all private providers) were identified as privately owned providers receiving financing from private equity investments at the group level. Through these investments, private equity companies controlled 36–76% of all group-level shares at the four focal private providers, thereby indirectly affecting 50 primary care centers (≈9% of all privately operated centers) and 387,317 primary care patients (≈9% of all privately listed patients). Table 3 displays the four focal providers, highlighting their respective intra-organizational levels and private equity investments, as well as the centers and patients affected by these investments.

**Table 3:** All private providers of primary care services in Sweden featuring private equity investments during 2024. The displayed percentages have been rounded

Multi-level actors	Private equity companies (percentages of all group-level shares controlled in multi-level actors)	Primary care centers (percentages of all private centers)	Primary care patients (percentages of all private patients)
Delivery level: Meliva Sverige Parent level: Meliva Group level: Mehiläinen Konserni	CVC Capital (56) LähiTapiola (20)	19 (3)	125,462 (3)
Delivery level: Kry Primärvård Parent level: Medical Supply Sweden Group level: Kry International	Creandum (11) Index Ventures (11) Accel London Investments (7) White Eye (7)	28 (5)	247,673 (5)
Delivery level: Min Doktor Parent level: MD International Group level: MD International	EQT Ventures (16) Swedbank Robur Ny Teknik (8) Carl Westin (7) Inbox Capital (2) Otiva (2) Human Xplore (1)	1 (0.2)	3,706 (0.08)
Delivery level: Trygg Hälsa Parent level: Trygg Hälsa Sverige Group level: Mindler	Luminar Ventures (14) Ventech Capital (13) Schibsted Tillväxtmedler (12) Back in Black Capital (11) Philian (4) LV Reldnim (4) 3T Invest (3)	2 (0.3)	10,476 (0.2)
*Total*	*Total*	*Total*	*Total*
N/A	N/A	50 (9)	387,317 (9)

**Source(s):** Authors' own work

## Discussion: understanding organizational diversity among Sweden's private providers

Throughout this article, our aim has been to develop a comprehensive description detailing the organizational diversity existing among private providers of primary care services in Sweden today. We studied such diversity across business types, core purposes, and external funding sources – three dimensions associated with ownership, intention and financing issues, respectively, that tend to influence the priorities of managers who lead organizations. Leveraging a dataset featuring all privately owned providers during 2024, our findings indicate there was little organizational diversity across the three management dimensions. In terms of business types, most private providers operated as private limited companies. Moreover, when it comes to core purposes, most privately owned providers conduct for-profit activities. And, finally, in terms of external funding sources, most private providers relied on credit institute loans.

We suggest our findings contribute to previous research on privately owned providers in Swedish primary care by offering an updated and expanded description of organizational diversity across key management dimensions that have hitherto only received sporadic and unsystematic attention from scholars. Our contribution ultimately facilitates a discussion where we draw on classical insights from institutional theory (DiMaggio and Powell, 1983; Meyer and Rowan, 1977) to conjecture that private providers in Sweden have augmented their proportions – while displaying little organizational diversity in their characteristics – as suppliers of primary care services during the 2010s and 2020s. The notion of isomorphism (Boxenbaum and Jonsson, 2017; DiMaggio and Powell, 1983) specifically highlights how organizations (e.g. private providers) may garner legitimacy from their stakeholders (e.g. politicians and financiers) by adopting dimensions (e.g. business types, core purposes and external funding sources) that align with normative expectations (or “institutionalized rules” (Meyer and Rowan, 1977, p. 340)) about what constitutes an adequate organization in a societal sector (e.g. Swedish primary care). When several or most organizations in a sector align with prevailing expectations, these organizations will, by extension, come to display little organizational diversity.

### Isomorphic patterns among privately owned providers

Our findings showing that, among private providers, private limited companies were the most common business type (encompassing ≈90% of all private providers, and affecting ≈80% of all privately operated centers and ≈75% of all privately listed patients during 2024) are quite similar to findings from previous research in Swedish primary care. Angelis *et al.* (2016), for instance, found that almost all privately owned providers of primary care services (≈95%) during 2014 operated as “private companies” – a category appearing to include private limited companies. We note Angelis *et al.* also showed that very few private providers (≈5%) operated as “foundations and economic associations,” thereby echoing our findings on foundations and economic associations. Andersson *et al.* (2021), moreover, found that almost all privately owned providers of primary care services (≈95%) during 2017 operated as “company groups” and/or “independent units” – two categories appearing to include private limited companies. Seen through an isomorphism lens, the dominance of private limited companies among privately owned providers in Swedish primary care would suggest important stakeholders, such as financiers, may be expecting these privately owned providers to operate as private limited companies today. Private limited companies are namely associated with resource requirements that offer financiers certain guarantees if the focal companies fail (Kaplan and Strömberg, 2009).

Despite considerable similarity across findings, our findings are, at times, challenging to compare with findings from previous research on private primary care providers in Sweden. These challenges surface because previous research is based on broader categorizations of business types than the categorizations we have utilized. In this sense, Angelis *et al.*’s (2016) private companies and Andersson *et al.*’s (2021) company groups could both include private limited companies and publicly traded companies. And Andersson *et al.*’s independent units could, in turn, include a range of proprietorships, partnerships and associations. Because our study uses more detailed categorizations of business types than previous research, we did not combine private limited and publicly traded companies; doing so would have hidden important ownership differences. Here, it is useful to underscore how ownership in private limited companies consists of shares that are privately bought and sold among select investors, whereas ownership in publicly traded companies consists of shares that are publicly bought and sold through stock exchanges (Armour *et al.*, 2017; de Jong, 2016). Such differences are, by extension, captured in the very notion of private limited companies as “private limited” and publicly traded companies as “publicly traded.”

To continue, our findings showing that, among private providers, conducting for-profit activities was the most frequent core purpose (encompassing ≈95% of all private providers, and affecting ≈95% of all privately operated centers and ≈95% of all privately listed patients during 2024) are consistent with findings from previous research in Swedish primary care. Previous findings show that, at separate time points during the 2010s, almost all private providers of primary care services in Sweden conducted for-profit activities (Häger Glenngård, 2023; Isaksson *et al.*, 2016; Vengberg *et al.*, 2021; Winblad *et al.*, 2021). We can, through our detailed categorization, see that the extensive presence of for-profit activities among privately owned providers is intertwined with the extensive presence of private limited companies – which conduct for-profit activities – throughout the 2010s and 2020s. And we can conversely see that the extensive presence of for-profit actors among private providers is also corroborated by the scarce presence of foundations – which conduct not-for-profit activities – throughout the 2010s and 2020s. This extensive intertwining of private limited companies and for-profit activities would, when seen through an isomorphism lens, indicate that stakeholders in Swedish primary care may nowadays be expecting privately owned providers to operate as private limited companies conducting for-profit activities.

The dominant presence of for-profit activities among privately owned providers is not unique to primary care; for-profit activities are present to a similar degree in Swedish eldercare (Broms *et al.*, 2024; Edlund *et al.*, 2025; Stolt *et al.*, 2011; Stolt and Winblad, 2009; Winblad *et al.*, 2017). The dominance of for-profit activities in Sweden's public sector has become fodder for recurring political debates (Hartman, 2011; Linarsson, 2017). For instance, heavy debates surrounded the so-called Reepalu investigation (Regeringskansliet, 2016), a mid-2010s governmental inquiry resulting in recommendations to introduce profit ceilings that initially generated distress among private providers of public services. Whereas such ceilings were subsequently discarded by the Swedish parliament, investigators were able to introduce heightened establishment requirements for private providers (Regeringskansliet, 2018). These requirements, together with certain capacity and administration requirements already contained in the LOU and LOV laws, eventually appear to have benefited private providers conducting for-profit activities. Providers conducting for-profit activities are often better financially equipped than providers conducting not-for-profit activities when it comes to meeting the requirements placed on private providers throughout Sweden's public sector (see Lindmark (2024) as well as Meagher and Szebehely (2013) for a similar argument in eldercare). Understanding what actors may benefit, and how they may benefit, from various requirements seems key to grasping the dominance of privately owned providers conducting for-profit activities in primary care services during 2024.

Lastly, our findings showing that, among private providers, credit institute loans were the most common external funding source (encompassing ≈21% of all private providers, and affecting ≈80% of all privately operated centers and ≈75% of all privately listed patients during 2024) are also challenging to compare with findings from previous research in Swedish primary care. These challenges arise because external funding sources have, to date, not been considered by researchers studying privately owned providers of primary care services in Sweden. Although our findings regarding external funding sources are difficult to compare with findings from previous research, we highlight that credit institute loans as external funding sources do not exhibit the same tendencies toward isomorphism today *vis-à-vis* private limited companies as business types and for-profit activities as core purposes. The presence of credit institute loans is, at least when it comes to private providers funded by such loans, not as dominant as the presence of private limited companies and for-profit activities. Among external funding sources, we also highlight that private equity investments – often perceived as controversial funding sources by citizens (cf. Svallfors and Tyllström, 2019) – were rather uncommon in Swedish primary care during 2024. Such investments were present among 2% of all private providers, which commanded approximately 10% of all privately operated centers and privately listed patients. Private equity investments were, in addition, present to different degrees (i.e. private equity companies controlled 36–76% of all group-level shares within the focal primary care providers), and these investments were almost always present alongside other external funding sources (e.g. credit institute loans).

Whereas private equity investments remain rather uncommon, it seems important to consider the relationships between private equity companies as financiers of primary care services and private providers as suppliers of primary care services. These relationships resemble a principal-agent separation between financiers and suppliers (cf. Kaplan and Strömberg, 2009), as private equity companies finance private providers to supply the actual primary care services. Such separation can generate management problems and, by extension, quality problems because service suppliers presumably possess more knowledge and expertise than service financiers about the conditions and complexities of primary care (Henry and Loomis, 2023; see Hoppania *et al.* (2024) for a similar argument in eldercare).

In pooling our findings with those stemming from previous research, we can, through the prism of primary care, see how market-inspired reforms have impacted Sweden's public sector over time. More than 10 years ago, the Swedish Competition Authority (2014, p. 39) claimed most private providers of primary care services were “small businesses” commanding one primary care center each. This claim remained true in 2024: while the largest private provider commanded 101 primary care centers, 70% of all private providers that year (including single-level actors and multi-level actors) commanded one primary care center, indicating most private providers were indeed “small businesses.” That said, aggregated findings throughout the 2010s and 2020s still suggest market-inspired reforms in Sweden have failed to attract a diversity of private providers into primary care. This failure is reflected in our findings showing that five conglomerate actors concentrated more than 50% of all privately operated centers and close to 60% of all privately listed patients during 2024. These conglomerate actors can be taken to display clear isomorphic patterns, considering all five were organized as private limited companies conducting for-profit activities and utilizing credit institute loans. This failure to attract diverse private providers thus presents patterns of concentration and isomorphism that may very well stifle competition and choice – two mechanisms hailed by politicians as pathways for cost savings and quality improvements in Swedish primary care (Ehn and Sundström, 2020; Hartman, 2011).

### Longitudinal and comparative approaches as future research avenues

We finish this article by reporting limitations that simultaneously offer future research avenues. One limitation pertains to our focus on a single year. Focusing on 2024, we mapped and analyzed all private providers in Swedish primary care to offer an updated and expanded description focusing on the organizational diversity displayed by these providers across three management dimensions. Such mapping and analysis inevitably engendered snapshot-like findings, and our strategy to devise a longitudinal perspective included juxtaposing and aggregating them with those deriving from previous research. However, we could not fully assess the three dimensions over time because previous research mostly features findings connected to core purposes. This is not surprising; researchers studying privately owned providers of primary care services in Sweden have addressed other topics than management dimensions. One future research avenue is thus to collect data on core purposes, business types and external funding sources at several time points – perhaps before and after select reforms like the LOU law or the LOV law were introduced. Such data collection would allow researchers to wholly examine how private providers of primary care services vary across multiple management dimensions in a longitudinal perspective.

Another limitation pertains to our reliance on a single context. Although Swedish primary care displays certain peculiarities, including particularly persistent market-inspired reforms, we propose it also features many aspects that make primary care in Sweden comparable to primary care in other Scandinavian contexts. Important aspects still permeating Swedish primary care – for example, full public reimbursement and considerable governmental involvement – can similarly be identified in public sectors across Scandinavia (Knutsson *et al.*, 2016; Vike *et al.*, 2024). Such similarities indicate our findings can fruitfully be extended beyond Sweden to grasp the characteristics of private providers throughout other Scandinavian countries with large public sectors. Still, another future research avenue is to gather primary care data on core purposes, business types and external funding sources throughout Scandinavia as a way of systematically examining how these three management dimensions may vary across several contexts.

We ultimately hope the two research avenues outlined above will inspire scholars to conduct longitudinal and/or comparative studies focusing on a significant, yet contentious, group of primary care providers combining private ownership and public reimbursement.
